# The Potential Role of Th9 Cell Related Cytokine and Transcription Factors in Patients with Hepatic Alveolar Echinococcosis

**DOI:** 10.1155/2015/895416

**Published:** 2015-10-05

**Authors:** Tuerhongjiang Tuxun, Shadike Apaer, Hai-Zhang Ma, Heng Zhang, Amina Aierken, Ren-Yong Lin, Hao Wen

**Affiliations:** ^1^Department of Liver & Laparoscopic Surgery, Digestive and Vascular Surgery Centre, First Affiliated Hospital of Xinjiang Medical University, Urumqi, Xinjiang Uyghur Autonomous Region 830054, China; ^2^State Key Laboratory Incubation Base of Xinjiang Major Diseases Research and Xinjiang Key Laboratory of Echinococcosis, First Affiliated Hospital of Xinjiang Medical University, Urumqi, Xinjiang Uyghur Autonomous Region 830054, China; ^3^Department of Ultrasonography, First Affiliated Hospital of Xinjiang Medical University, Urumqi, Xinjiang Uyghur Autonomous Region 830054, China

## Abstract

Human alveolar echinococcosis (AE) is a lethal parasitic infectious disease which may lead to liver failure if left untreated. It is caused by the larval stage of the fox tapeworm *Echinococcus multilocularis* and usually develops a substantial infiltrative occupation in solid organs. During the infection, T helper subsets are known to play crucial role in crosstalk between the parasite and human host. Th9 cells, a new member of CD4^+^ T cell family which is characterized by its specific cytokine IL-9 and transcription factors PU.1 and IRF-4, have been known recently to have a critical role in allergic diseases, and cancers as well as the parasitic infection. To assess the potential role of Th9 cells during the infection, the mRNA levels of IL-9, PU.1, and IRF-4 both in peripheral blood mononuclear cells and in liver tissues were, respectively, detected by using real-time PCR. The plasma concentration levels of IL-9 were detected by using enzyme linked immunosorbent assay (ELISA). Th9 related cytokine IL-9 and transcription factors PU.1 and IRF-4 mRNA levels elevated both in PBMCs, and in hepatic lesion and paralesion tissues in AE patients. This may facilitate the infiltrative growth of the parasite and its persistence in human host.

## 1. Introduction

Human alveolar echinococcosis (AE) is a lethal parasitic infectious disease which may lead to liver failure if left untreated. It is caused by the larval stage of the fox tapeworm* Echinococcus multilocularis* and usually develops a substantial occupation in solid organs [1]. Liver is the most common targeted organ which accounts for nearly 95% of infections, with 5% percent to other organs including lung, brain, and bone. The parasite usually displays a cancer-like biological behavior and infiltrates to the liver tissue and major vasculature and is characterized by its granulomas formation. Previous studies have shown that CD4^+^ T cell subsets play an active role in the development and immune response in AE and showed an upregulation of T helper (Th) 2 immune response which has been shown in the liver lesions locally and in circulating lymphocytes in animal models and patients. There is, however, evidence that cellular immunity and Th1 related cytokines also play a role by controlling totally the parasite growth in some individuals and limiting the size of the lesions in patients with the diseases. Clearly, Th1/Th2 imbalance plays an important role in controlling the immunopathogenesis of AE infection. Meanwhile, the immunological responses of host's Th17 and Treg cells against AE have been extensively studied and displayed an important role of both T helper cells in immune tolerance and immune-pathological injury during the infection [2, 3].

Th9 cells, a new member of CD4^+^ T cell family which is characterized by its specific cytokine IL-9 and transcription factors PU.1 and IRF-4, have been known recently to have a critical role in allergic diseases and cancers as well as the parasitic infection. Our previous study has shown that Th9 cells have been involved in the immune response on cystic echinococcosis [4]. However, whether this novel T cell subset is involved in regulating the immune response during the infection and development of AE is still unclear. Thus, we aimed to evaluate mRNA expression levels of IL-9, PU.1, and IRF-4 in PBMCs and circulating cytokine IL-9 in AE patients and healthy control, as well as the mRNA levels of IL-9, PU.1, and IRF-4 in lesion, paralesion, and normal hepatic tissues in hepatic AE patients in an effort to evaluate their potential role, if any, in the disease and their relation to disease activity.

## 2. Materials and Methods

### 2.1. Patients

This study was conducted in accordance with the Declaration of Helsinki (1964) and conformed to the approved institutional guidelines and was approved by the Ethical Committee of Xinjiang Medical University. Twenty-eight subjects were eligible for this study and informed consents were obtained from every single subject. They were divided into two groups: healthy control group (HC) and alveolar echinococcosis group (AE), with each group containing fourteen subjects. Healthy controls were qualified for medical testing and attended voluntarily, including 8 men and 6 women. All the patients with AE infection including 6 men and 8 women underwent surgery in the Department of Liver & Laparoscopic Surgery, First Affiliated Hospital of Xinjiang Medical University. The infection with* E. multilocularis* in all patients was confirmed by postoperative pathological examination.

All AE patients were classified according to the WHO/IWGE classification [5]. No patient was treated with anti-inflammatory drugs such as nonsteroidal anti-inflammatory drugs and corticosteroids. None had chronic inflammatory disease, cardiovascular disease, disseminated intravascular coagulation, advanced lung disease, renal failure, malignant disease, or other infectious diseases (such as septicemia and pneumonia).

### 2.2. Blood Samples

Blood samples were obtained from all the subjects in the recumbent position with a 21-gauge needle for clean venipuncture of an antecubital vein after admission in a fasting state on the following morning of the admission day. The samples were collected into collection tubes containing 0.2 mL sodium heparin. Peripheral blood mononuclear cells (PBMCs) were prepared by Ficoll density gradient for analysis of real-time polymerase chain reaction (PCR). Plasma was obtained after centrifugation and stored at −80°C for the measurement of the cytokine and transcription factors.

### 2.3. Surgery and Liver Tissue Samples

All resections were performed via laparotomy. All surgical procedures were performed by three experienced hepatic surgeons. Experienced senior surgeons carried out all partial hepatic resections to guarantee at least one-centimeter safety margin. Frozen section examinations at the hepatic transsection line were performed in all cases.

All surgical specimens were reviewed by a senior pathologist. Clinical and pathologic staging were reassessed according to the World Health Organization on alveolar echinococcosis PNM classification of AE. Only one sample of lesion, paralesion (hepatic tissue within 1 cm from the AE lesion), and normal liver tissues was obtained for all the patients. The specimens had been snap-frozen in RNA later and liquid nitrogen for 15 seconds and were immediately stored at −80°C.

### 2.4. IL-9, PU.1, and IRF-4 mRNA Expression Determined by Real-Time PCR

Total RNA was extracted with TRIzol extraction (Invitrogen) according to the manufacturer's instruction. cDNA was synthesized using PrimerScript RT reagent kit (Takara, Biotechnology, Dalian, China). SYBR Green primers for human IL-9, PU.1, IRF-4, and GAPDH mRNA were purchased from Sangon, Shanghai. The samples were analyzed using the ABI Prism 7900 Sequence Detection System (BioRad, Life Science Research, Hercules, CA, USA). The following primer pairs were used: IL-9: F: 5′-CTCTAGCAGTCCACT TCACCAA-3′, R: 5′-ACAGCATGGGTCTGTCTTCT-3′; PU.1: F: 5′-GGAGCCCGGCTGGATGTTAC-3′, R: 5′-CACCAGGTCTTCTGATGGCTGA-3′; IRF-4: F: 5′-GACCCGCAGATGTCCATGAG-3′, R: 5′-TGTAGTTGTGAACCTGCTGGG-3′; GAPDH: F: 5′-GCACCGTCAAGGCTGAGAAC-3′, R: 5′-TGGTGAAGACGCCAGTGGA-3′. For each sample, mRNA expression was normalized to the level of GAPDH housekeeping genes (Table 1).

Both the housekeeping and the target genes for each sample were amplified in triplicate using the following procedure of initial denaturation at 95°C for 3 min, 39 cycles of 95°C for 10 s, 60°C for 30 s, and 65°C for 5 s, and after that they directly evolved to the ultimate temperature of 95°C. The 2^−ΔΔCt^ method was used to determine the cycle number Ct value corresponding to a specific fluorescence threshold and quantify the target genes.

### 2.5. ELISA Detection of Plasma Concentration Level of IL-9

The plasma levels of IL-9 were measured by enzyme-linked immunosorbent assay (ELISA), following the manufacturer's protocols (all kits from Elabscience Biotechnology Co., Ltd.). The minimal detectable concentrations range was 15.625 pg/mL for IL-9, and the sensitivity was 9.375 pg/mL. Intra-assay and interassay coefficients of variation for all ELISA were <5% and <10%, respectively. All samples were measured in duplicate.

### 2.6. Statistical Analysis

Statistical analysis was performed using the SPSS 17.0, and values are expressed as median (P_25_ and P_75_) in the text and tables. The data were analyzed by using nonparametric Friedman test. If significance was found, Wilcoxon signed ranks test and Mann-Whitney *U* test were performed to detect the difference among groups. Spearman correlation was used as a test of correlation between two continuous variables. Correlations were determined by Spearman correlation coefficients. A probable value of *P* < 0.05 was considered to be statistically significant.

## 3. Results

### 3.1. Basic Clinical Characteristics of Patients

Age, gender, diameters of lesions, and previous surgical history are listed in Table 2. There were no significant differences between the two groups. Four patients have undergone laparotomy for cholecystectomy in three patients and laparoscopic exploration in one patient at another institution. All patients in the AE group underwent hepatic resections. All AE patients were classified according to the PNM classification of WHO/IWGE as abovementioned. Among them, 12 patients were classified as P1N0M0 and the rest of them were classified as P2N0M0 (P1: peripheral hepatic lesion with no proximal hepatic vascular or biliary involvement; P2: central hepatic lesion with proximal involvement of vessels biliary ducts in one lobe; N0: no regional involvement; M0: no metastasis).

### 3.2. Expression of IL-9, PU.1, and IRF-4 mRNA in PBMCs in Two Groups

**Figure 1:**
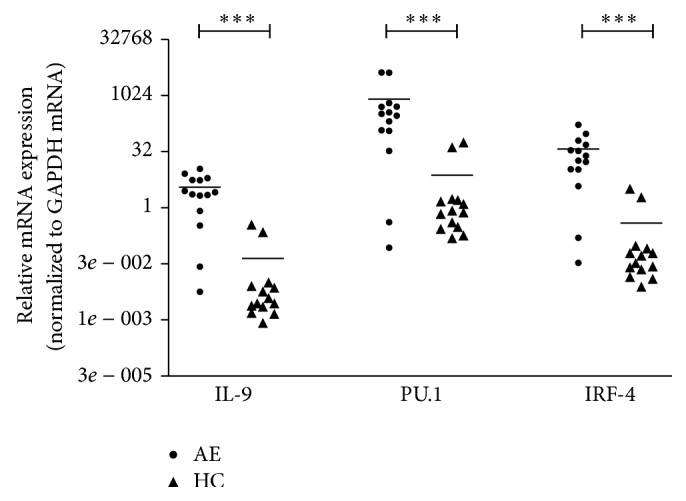
The expression levels of cytokine and transcription factors in PBMCs in AE and HC group. AE: alveolar echinococcosis; HC: healthy control. *P* value < 0.001 with marker ∗∗∗; *P* value 0.001 to 0.01 with marker ∗∗; *P* value 0.01 to 0.05 with marker ∗; *P* value > 0.05 is not significant.

IL-9 is the main cytokine secreted by Th9 cells. PU.1 and IRF-4 are significant transcription factors for the differentiation of Th9 cells. We thus investigated the expressions of IL-9, PU.1, and IRF-4 mRNA in PBMCs from patients with AE and healthy controls. As shown in Figure 1 and Table 3, the expression levels of IL-9, PU.1, and IRF-4 mRNA were all significantly higher in AE group than those in HC group (^***^ *P* < 0.001, ^***^ *P* < 0.001, and ^***^ *P* < 0.001, resp.).

### 3.3. Expression of IL-9, PU.1, and IRF-4 mRNA in Different Liver Tissues of AE Patients

**Figure 2:**
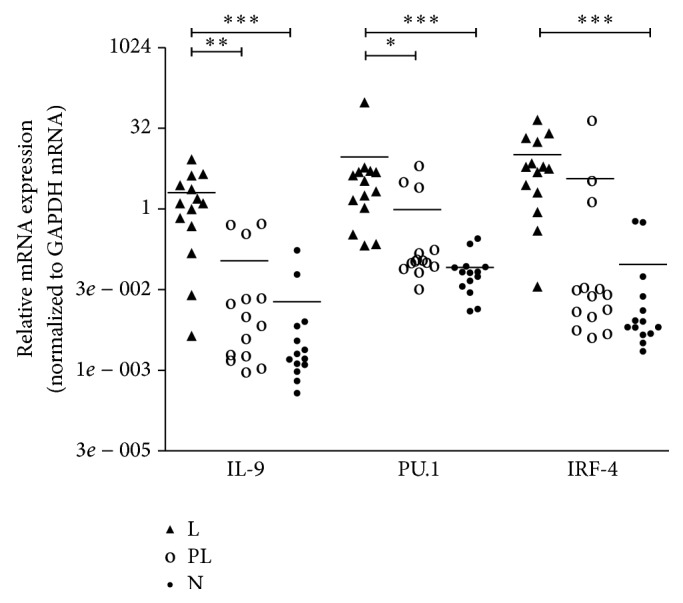
The expression levels of cytokine and transcription factors in different liver tissues. L: lesion group; PL: paralesion group; N: normal liver tissues. *P* value < 0.001 with marker ∗∗∗; *P* value 0.001 to 0.01 with marker ∗∗; *P* value 0.01 to 0.05 with marker ∗.

As shown in Figure 2, the relative expression levels of IL-9 mRNA in lesion tissues (1.248; IQR, 0.389–3.071) were markedly higher than those in paralesion and normal tissues (0.008; IQR, 0.002–0.105; ^**^ *P* = 0.002, 0.002; IQR, 0.001–0.007; ^***^ *P* < 0.001), and there was no statistical significance between paralesion group and normal group (*P* > 0.05). PU.1 mRNA expression levels were elevated in lesion tissues (2.675; IQR, 0.851–4.865) as compared with those in paralesion and normal tissues (0.111; IQR, 0.085–0.782; ^*^ *P* = 0.013, 0.065; IQR, 0.034–0.083; ^***^ *P* < 0.001). Significantly elevated levels of IRF-4 mRNA were found in lesion group (0.025; IQR, 0.009–0.413) compared to those in normal group (0.007; IQR, 0.005–0.031; ^***^ *P* < 0.001). However, there was no statistical significance between lesion group and paralesion group (*P* > 0.05).

### 3.4. Plasma Cytokine Concentrations in Two Groups

**Figure 3:**
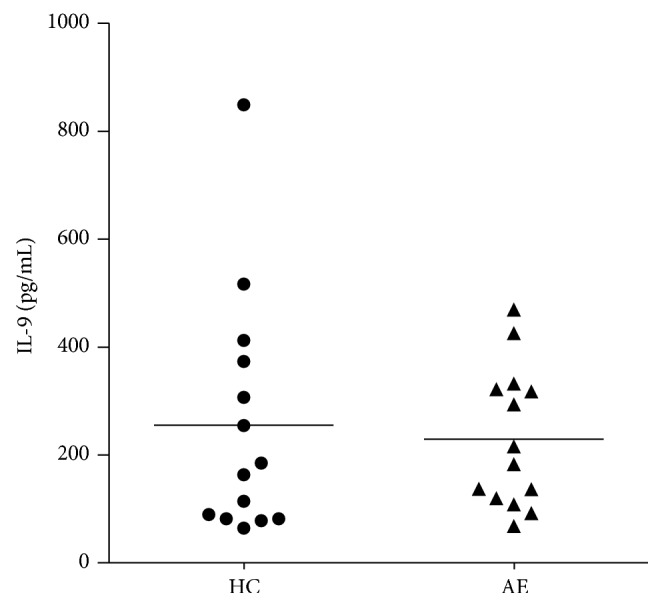
The plasma IL-9 concentration levels in AE and HC group. IL-9 concentrations in AE group were slightly lower than those in HC group, and there was no statistical significance found between the two groups (*P* > 0.05). HC: healthy control; AE: alveolar echinococcosis.

As shown in Figure 3, the plasma IL-9 concentration levels were slightly lower in AE group than those in HC group, and there was no statistical significance between the two groups (*P* > 0.05).

### 3.5. Correlation Analysis between IL-9 and PU.1 and IRF-4 mRNA Expression Levels in PBMCs and Liver Tissues

**Figure 4:**
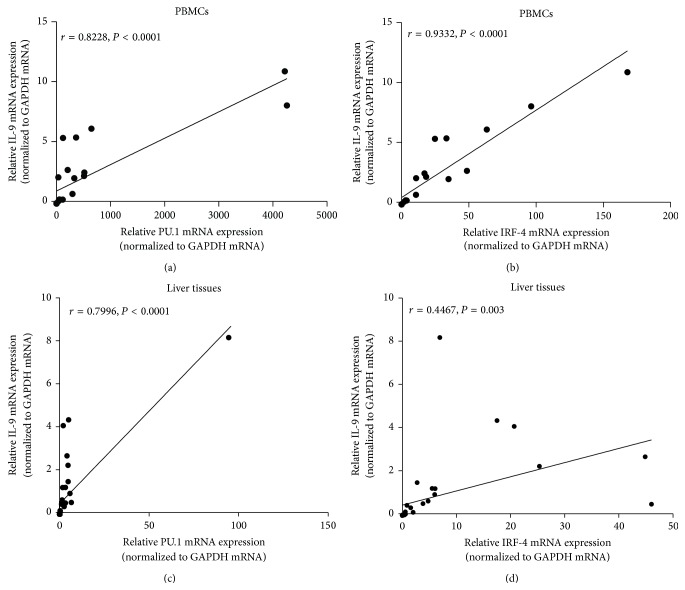
Spearman correlation of Th9 cells cytokine and transcription factors in PBMCs and liver tissues. ((a) and (b)) IL-9 mRNA expressions in PBMCs were correlated positively both with PU.1 and with IRF-4 mRNA expressions (*r* = 0.8228, *P* < 0.0001; *r* = 0.9332, *P* < 0.001, resp.). ((c) and (d)) The expression of IL-9 mRNA in liver tissues had a positive correlation with both PU.1 and IRF-4 mRNA expression (*r* = 0.7996, *P* < 0.0001; *r* = 0.4467, *P* = 0.003, resp.).

As showed in Figures 4(a) and 4(b), IL-9 mRNA expression in PBMCs had a positive correlation with PU.1 and IRF-4 mRNA expression (*r* = 0.8228, ^***^ *P* < 0.0001; *r* = 0.9332, ^***^ *P* < 0.0001, resp.). Spearman correlation coefficients indicated that IL-9 mRNA expression in the liver tissues had a positive correlation with both PU.1 and IRF-4 mRNA expression (*r* = 0.7996, ^***^ *P* < 0.0001, *r* = 0.4467; ^**^ *P* = 0.003, resp.) as shown in Figures 4(c) and 4(d).

## 4. Discussion

Along the years, the important role of both innate and acquired immunity in the field of* E. multilocularis* infection has been extensively studied. Among them, the role of CD4^+^ T cells in the development of the parasitic lesion and immune evasion from human immune system's attack has been highlighted [6]. It is widely acknowledged that the parasitic infection can initiate a successful crosstalk between human immune system and parasite itself and actively modulate CD4^+^ T cells development, thus guaranteeing its persistent survival. Both clinical and experimental studies displayed that* E. multilocularis* infection accompanies the imbalanced Th1/Th2 immune profile which is in favor of the parasitic survival [7–9]. Although different infection phase demonstrated various immune response patterns, the enhanced Th2 response and ameliorated Th1 response are considered to be linked with the survival of the parasite. Recent advances in the field of immunology have commenced new era for parasitic immunology. Further studies on novel T cell subset have unveiled the previously unknown phenomenon. Like other autoimmune diseases, cancers, fungal diseases, and parasitic infections, the reciprocal role of Th17 and Treg cells has drawn a great interest [10, 11]. Our previous animal and human studies have shown that the Treg cells overwhelmed immune response, although no significant alteration was found in terms of Th17 [2, 12]. As a new member of CD4^+^ T cell family, Th9 cells have been known to play a significant role in some diseases, such as allergic diseases and cancers as well as the parasitic infections [13]. Furthermore, Th9 cells, through the expression of the effector cytokine IL-9, are more efficient than classical Th2 cells at orchestrating antihelminthic responses in helminthic infection in vivo [14]. Our recent research has shown that Th9 and its functional cytokine IL-9 are upregulated in CE patients suggesting that they may be involved in regulating the immune response or even promote the process of* Echinococcus granulosus* infection [4]. However, whether Th9 cells also participate in the AE infection has not been studied yet, so we implemented this research to investigate its potential function in the AE infection.

Two decades before the description of Th9 cells, it was reported that IL-9 production by CD4^+^ T cells depends on IL-2, is promoted by IL-4 and transforming growth factor TGF-*β*, and is further enhanced by IL-1, while IFN-*γ* represents a potent inhibitor of IL-9 expression [15]. As a great body of previous studies have revealed that IL-9 produced by Th9 cell promoted pathogenic processes in several autoimmune diseases, such as allergy and asthma [16]. Accumulating studies revealed a major role for IL-9 in protection against and expulsion of some helminthic parasites. As demonstrated in a recent study, IL-9 blockade prevents worm expulsion in mice infected with* Trichinella spiralis*, indicating that IL-9 was a pivotal cytokine to mediate effective expulsion of* T. spiralis* and was important for mediating an effective worm clearance [17]. In this study, IL-9 mRNA expression level was markedly increased in AE patient's PBMCs and, most intriguingly, the IL-9 mRNA expression levels were exponentially higher in AE lesions than in paralesion and normal liver tissues. However, the plasma IL-9 concentration levels in patients with AE were lower than in HC group without statistical significance. This suggests that IL-9 may be involved in AE infection process and may have a potential role in parasite clearance as shown in previous studies [4].

PU.1 and another transcription factor IRF-4 are key transcription factors that induce differentiation of Th0 cells into Th9 cells. PU.1 plays a crucial role in the regulation and expression of CD80, CD86, and cells in the hematopoietic system and it can also block several autoimmune diseases and even induce donor-specific tolerance to allograft [18]. A recent research showed that modifications in the PU.1 promoter uniquely and dynamically control the threshold for Th9 cells development in naive and memory CD4^+^ T cells [19]. Detailed analyses revealed that some cytokines play a pivotal role in Th9 development and function by inducing the expression of PU.1 [20]. Some studies have described PU.1 as a unique regulator of Th9 memory acquisition and Th9 immunity [21]. It has been reported to determine Th9 cells development and can enhance or be required for IL-9 production in Th9 cells [22]. T cell specific deletion of PU.1 results in decreased IL-9 production in vitro, as well as in vivo, coincident with diminished allergic inflammation [23]. IRF-4 was also found to be crucial in Th9 response, as IRF-4 deficient CD4^+^ T cell failed to develop into IL-9-producing Th9 cells, and IRF-4 RNAi knockdown suppressed IL-9 production in WT CD4^+^ T cells. Furthermore, chromatin immunoprecipitation analysis indicated that IRF-4 directly bound to the IL-9 promoter in Th9 cells [24].

Our previous study on cystic echinococcosis, which is caused by* E. granulosus*, has showed a distinct increase of both IL-9 and PU.I in infected patients' PBMCs [4]. In line with this, this study for the first time displayed the dramatic highly elevated levels of IL-9, PU.1, and IRF-4 mRNA in both PBMCs and hepatic tissues, albeit with no clear alteration in plasma levels. Hepatic AE patients in this study were all categorized according to the PNM classification of WHO/IWGE and 12 were categorized P1N0M0 and two were P2N0M0. All the cases were progressive AE patients. The regional discrepancies of IL-9, PU.1, and IRF-4 mRNA are quite clear in parasitic lesion, paralesion, and normal tissues. This may be because the parasite itself and its functional molecules modulate the systemic and regional T helper cell differentiation thus creating Th9 dominating immune response which is now known to be in favor of parasitic survival and, thus, may play a role in immune response and granuloma formation during the AE infection. Furthermore, as revealed in the correlation analysis in this study, IL-9 expression levels were positively correlated with the levels of PU.1 and IRF-4 expression, respectively, both in PBMCs and in local hepatic lesion tissues. These details further confirmed the significant roles of PU.1 and IFR-4 in the differentiation and function of Th9 cells.

In conclusion, the results reveal a rising trend in Th9 levels in both local and PBMCs in patients with AE. Moreover, a recent study implicated a potential role of Th9 cells promoting pathology in filarial disease and suggested it is dependent on the effect of IL-9 on promoting inflammatory responses in target cells [25]. These details suggest that Th9/IL-9 were involved in the AE infection and response during the* E. multilocularis*-host interplay and might play critical potentially beneficial roles in the parasite survival and pathogenesis in AE infection.

In our study, we disclosed for the first time that Th9 cell and its signature cytokine IL-9 and transcription factors PU.1 and IRF-4 were upregulated in peripheral and local hepatic tissues in AE patients. Our research strongly indicates that Th9/IL-9 may be associated with development of* E. multilocularis* infection and even may play a critical potential role to facilitate the infiltrative growth of the parasite and its persistence in human host. Further studies should be implemented for exploring the exact mechanism, and we believe it will deserve further attention in future studies.

## Figures and Tables

**Table 1:** Primer sequence and amplicon of GAPDH, IL-9, PU.1, and IRF-4.

Gene	Sequences	Tm (°C)	Amplicon (bp)
GAPDH	F: 5′-GCACCGTCAAGGCTGAGAAC-3′	62.10	267
	R: 5′-TGGTGAAGACGCCAGTGGA-3′	61.70	

IL-9	F: 5′-CTCTAGCAGTCCACTTCACCAA-3′	59.70	112
	R: 5′-ACAGCATGGGTCTGTCTTCT-3′	58.64	

PU.1	F: 5′-GGAGCCCGGCTGGATGTTAC-3′	62.94	79
	R: 5′-CACCAGGTCTTCTGATGGCTGA-3′	62.00	

IRF-4	F: 5′-GACCCGCAGATGTCCATGAG-3′	60.53	82
	R: 5′-TGTAGTTGTGAACCTGCTGGG-3′	60.20	

*Note*. F: forward; R: reverse.

**Table 2:** Basic clinical characteristics of patients with hepatic alveolar echinococcosis.

Characteristics	HC	AE
	(*n* = 14)	(*n* = 14)
Age (years)	36.5 (25.75–41.00)	38.0 (24.75–42.50)
Sex (male/female)	8 : 6	6 : 8
Location (right/left)	*N*	12 : 2
^*^Diameter of lesions (cm)	0	10.37 (7.9–14.21)
Previous operation	0 (14)	4 (14)

Values are expressed as median (P_25_ and P_75_) or number; HC: healthy control; AE: alveolar echinococcosis;  ^*^for patient with multilesion, the average sizes were applied.

**Table 3:** The expression levels of IL-9, PU.1, and IRF-4 mRNA in PBMC in two groups.

	HC	AE
	(*n* = 14)	(*n* = 14)
IL-9/GAPDH	0.003	2.449
(copies/copies)	(0.002–0.008)	(0.693–5.706)^***^

PU.1/GAPDH	0.771	313.105
(copies/copies)	(0.288–1.591)	(94.806–548.131)^***^

IRF-4/GAPDH	0.0413	21.583
(copies/copies)	(0.0196–0.082)	(8.954–52.310)^***^

Values are expressed as median (P_25_ and P_75_); HC: healthy control; AE: alveolar echinococcosis.

## References

[1] Bresson-Hadni S., Beurton I., Bartholomot B. (1998). Alveolar echinococcosis. *Hepatology*.

[2] Tuxun T., Wang J.-H., Lin R.-Y. (2012). Th17/Treg imbalance in patients with liver cystic echinococcosis. *Parasite Immunology*.

[3] Lechner C. J., Grüner B., Huang X., Hoffmann W. H., Kern P., Soboslay P. T. (2012). Parasite-specific IL-17-type cytokine responses and soluble IL-17 receptor levels in Alveolar Echinococcosis patients. *Clinical and Developmental Immunology*.

[4] Pang N., Zhang F., Ma X. (2014). Th9/IL-9 profile in human echinococcosis: their involvement in immune response during infection by *Echinococcus granulosus*. *Mediators of Inflammation*.

[5] Kern P., Wen H., Sato N. (2006). WHO classification of alveolar echinococcosis: principles and application. *Parasitology International*.

[6] Vuitton D. A., Gottstein B. (2010). *Echinococcus multilocularis* and its intermediate host: a model of parasite-host interplay. *Journal of Biomedicine and Biotechnology*.

[7] Riganò R., Buttari B., De Falco E. (2004). Echinococcus granulosus-specific T-cell lines derived from patients at various clinical stages of cystic echinococcosis. *Parasite Immunology*.

[8] Kocherscheidt L., Flakowski A.-K., Grüner B. (2008). Echinococcus multilocularis: inflammatory and regulatory chemokine responses in patients with progressive, stable and cured alveolar echinococcosis. *Experimental Parasitology*.

[9] Liance M., Bresson-Hadni S., Meyer J. P., Houin R., Vuitton D. A. (1990). Cellular immunity in experimental Echinococcus multilocularis infection. I. Sequential and comparative study of specific in vivo delayed-type hypersensitivity against E. multilocularis antigens in resistant and sensitive mice. *Clinical and Experimental Immunology*.

[10] Sakaguchi S., Ono M., Setoguchi R. (2006). Foxp3^+^CD25^+^CD4^+^ natural regulatory T cells in dominant self-tolerance and autoimmune disease. *Immunological Reviews*.

[11] Mucida D., Park Y., Kim G. (2007). Reciprocal TH17 and regulatory T cell differentiation mediated by retinoic acid. *Science*.

[12] Wang J., Lin R., Zhang W. (2014). Transcriptional profiles of cytokine/chemokine factors of immune cell-homing to the parasitic lesions: a comprehensive one-year course study in the liver of E. multilocularis-infected mice. *PLoS ONE*.

[13] Jia L., Wu C. (2014). Differentiation, regulation and function of th9 cells. *T Helper Cell Differentiation and Their Function*.

[14] Licona-Limón P., Henao-Mejia J., Temann A. U. (2013). Th9 cells drive host immunity against gastrointestinal worm infection. *Immunity*.

[15] Veldhoen M., Uyttenhove C., van Snick J. (2008). Transforming growth factor-*β* “reprograms” the differentiation of T helper 2 cells and promotes an interleukin 9-producing subset. *Nature Immunology*.

[16] Schmitt E., Klein M., Bopp T. (2014). Th9 cells, new players in adaptive immunity. *Trends in Immunology*.

[17] Angkasekwinai P., Srimanote P., Wang Y.-H. (2013). Interleukin-25 (IL-25) promotes efficient protective immunity against Trichinella spiralis infection by enhancing the antigen-specific IL-9 response. *Infection and Immunity*.

[18] Xiang J., Gu X., Qian S., Chen Z. (2008). Graded function of CD80 and CD86 in initiation of T-cell immune response and cardiac allograft survival. *Transplant International*.

[19] Ramming A., Druzd D., Leipe J., Schulze-Koops H., Skapenko A. (2012). Maturation-related histone modifications in the PU.1 promoter regulate Th9-cell development. *Blood*.

[20] Goswami R., Jabeen R., Yagi R. (2012). STAT6-dependent regulation of Th9 development. *The Journal of Immunology*.

[21] Gerlach K., Hwang Y., Nikolaev A. (2014). TH9 cells that express the transcription factor PU.1 drive T cell-mediated colitis via IL-9 receptor signaling in intestinal epithelial cells. *Nature Immunology*.

[22] Chang H.-C., Sehra S., Goswami R. (2010). The transcription factor PU.1 is required for the development of IL-9-producing T cells and allergic inflammation. *Nature Immunology*.

[23] Goswami R., Kaplan M. H. (2012). Gcn5 is required for PU.1-dependent IL-9 induction in Th9 cells. *The Journal of Immunology*.

[24] Staudt V., Bothur E., Klein M. (2010). Interferon-regulatory factor 4 is essential for the developmental program of T helper 9 cells. *Immunity*.

[25] Anuradha R., George P. J., Hanna L. E. (2013). IL-4-, TGF-beta-, and IL-1-dependent expansion of parasite antigen-specific Th9 cells is associated with clinical pathology in human lymphatic filariasis. *The Journal of Immunology*.

